# Clinical manifestations and multi-slice computed tomography characteristics of solid pseudopapillary neoplasms of the pancreas between males and females

**DOI:** 10.1186/s12880-019-0390-9

**Published:** 2019-11-12

**Authors:** Shuguang Shi, Ying Zhou, Chunhong Hu

**Affiliations:** 1 Medical Imaging Center, the First Affiliated Hospital of Soochow University, No.188 Shizi Street, Suzhou, 215006 Jiangsu China; 2 Department of Radiology, First people’s Hospital of Lianyungang, Lianyungang, China

**Keywords:** Pancreatic tumor, Tomography, X-ray computed, Solid pseudo-papillary neoplasm of pancreas, Male

## Abstract

**Background:**

Solid-pseudo papillary neoplasms of pancreas (SPNP) are rare in men and are often misdiagnosed. This study aimed to analyze the clinical and multi-slice computer tomography (MSCT) features of patients with SPNP, and examine the differences between males and females.

**Methods:**

In this retrospective cohort study, the clinical and imaging data of 29 patients with histolopathologically confirmed SPNP (seven males and 22 females) that underwent radical resection, and underwent preoperative MSCT at the First People’s Hospital of Lianyungang between August 2010 and December 2018 were collected. All MSCT images were reviewed by two radiologists; disagreements were ruled by a third one.

**Results:**

The median age of the 29 patients with SPNP was 30 (range, 12–70) years. The male patients were older than the female patients [median, 56 (28–66) vs. 29 (12–70), *P* = 0.012]. The median tumor size was 3.9 (range, 2.0–6.4) cm in the male SPNP patients, which was significantly lower than the 7.0 (range, 4.6–14.6) cm in the female patients (*P* < 0.001). The calcification rate of the SPNP was significantly higher in male than in female patients (*P* = 0.013). The percentage of solid tumor was higher in males than in females (*P* = 0.036). Capsule, bleeding, and enhancement in the arterial and venous phases were not significantly different between the male and female patients (all *P* > 0.05).

**Conclusion:**

The imaging features of male SPNP are distinct from those of female patients. In males with pancreatic lesions, MSCT generally shows relatively small lesions with higher percentages of solid components and calcification, with typical enhancement suggesting SPNP.

## Background

Solid-pseudo papillary tumor of pancreas (SPNP) is a rare pancreatic tumor that mainly occurs in young women with the ages of 20–30 years old. Indeed, more than 90% of the cases are in females and 85% are in females < 30 years of age [1]. SPNP is an indolent tumor and its prognosis is very good, with reported 5-year survival of 95–100% [1, 2]. An exceptional feature of SPNP is that it is rare in males. In fact, SPNP is not even considered as a differential diagnosis in men with pancreas lesions, leading to inaccurate preoperative diagnosis, staging, and prognosis [3–5]. Nevertheless, it has been suggested that SPNP could be more aggressive in men than in women [6–11], but the rarity of the disease in men limits the data regarding them. SPNP is a rare benign or lowly malignant tumor that originate from pancreatic pluripotent stem cells [12], and accounts for about 0.3–2.7% of all pancreatic tumors. The pathogenesis of SPNP is unclear, but it has been suggested that the development of SPNP is associated with estrogen [13].It has been reported that tumor stroma and gene mutations exist gender difference [14]. Some researchers suggested that the malignancy of SPNP is associated with the mitosis rate of the tumor cells, as well as nuclear atypia [15].

Most previous studies considered the clinical and pathological characteristics of SPNP; only one study examined the computed tomography (CT) features pf SPNP between males and females [3]. At imaging, the differential diagnosis in male patients should include pancreatic cancer, pancreatic pseudocyst, and nonfunctional neuroendocrine tumor.

Improving our knowledge about the imaging features of SPNP could improve its diagnosis in male patients. Ever improving technologies could help achieve this goal. Therefore, the aim of this retrospective study was to analyze the clinical and multi-slice computed tomography (MSCT) data of patients with SPNP according to sex. The differences in the clinical manifestations and MSCT characteristics of the male and female patients were compared to improve the understandings of SPNP and help increasing the diagnostic accuracy.

## Methods

### Study design and patients

In this retrospective cohort study, the clinical and imaging data of patients with histopathologically confirmed SPNP that underwent radical resection and preoperative MSCT at the First People’s Hospital of Lianyungang between August 2010 and December 2018 were collected. This study was approved by the Ethics Committee of the First People’s Hospital of Lianyungang. The need for individual consent was waived by the committee.

The inclusion criteria were: 1) the MSCT confirming the lesion was done at the First People’s Hospital of Lianyungang; 2) treatment-naïve before the radical operation; 3) available complete clinical, pathological, and imaging data; and 4) treatments were conducted < 1 month after MSCT. The exclusion criteria were: 1) underwent plain CT scanning only; 2) received other treatments before operation; 3) imaging data were unavailable; or 4) the time from examination to treatments was too long.

### Examinations

All 29 patients underwent preoperative plain and enhanced MSCT scanning after an 8-h fast, using a VCT 64-row spiral CT scanner (GE Healthcare, Waukesha, WI, USA). At 30 min before scanning, the patients were asked to drink 800 ml of water to induce the dilation of the gastric cavity and duodenum, and to drink 250–300 ml of water immediately before scanning. The patients were placed in supine position and asked to hold their breath after inhalation. After plain scanning was conducted, a high pressure injector was used to inject the nonionic iodine contrast agent (ultravist; 80–100 ml, 1.5 ml/kg) through the forearm cubital vein, at 3.0 ml/s. Scanning was conducted at 25 s (arterial phase, AP), 60 s (portal venous phase, VP), and 90 s (delayed phase, DP), covering the area from the diaphragmatic dome to the inferior pole of bilateral kidneys. The scanning parameters were: tube voltage of 120 kV, tube current of 280–300 mAs, screw pitch of 1.0, layer thickness of 5 mm, and interlayer spacing of 5 mm. During reconstruction, layer thickness was 1 mm, and interlayer spacing was 0.8 mm. All the original data were input to the ADW4.6 workstation (GE Healthcare, Waukesha, WI, USA) for multiplanar reconstruction.

### Image analysis

All images were reviewed by at least two senior radiologists (attending radiologists or higher) with at least 10 years’ experience. Disagreements were solved by discussion with a third reviewer, who was a chief radiologist. The CT values of the lesions were measured at the solid components at the same sites, with the area of the region of interest (ROI) being ≥0.3 cm^2^. The lesion sites (head, neck, body, and tail of pancreas), size (maximal diameter), calcification (yes or no), bleeding (yes or no), capsule (yes or no), internal component (solid tumor: the tumor was mainly composed of solid components, with small amount of cystic changes, and the cystic component was < 10%; cystic and solid tumor; and cystic tumor), density, enhancement mode, changes of the pancreatic ducts, pancreas atrophy, retroperitoneal lymph nodes, and changes of the other organs were comprehensively examined and analyzed. The imaging results were compared with the surgical findings and pathological results. The enhancement degrees of the tumors were assessed by enhanced scanning, and the CT values of the solid components of SPNP in the plain phase (PP), AP, and VP were measured. The enhancement degrees were classified into high enhancement (enhancement higher than that of pancreas), moderate enhancement (enhancement similar to that of pancreas), and low enhancement (enhancement lower than that of pancreas). The absolute enhancement value of the solid component of SPNP in AP was calculated as follow: A = AP – PP, while the value in VP was calculated as follow: V = VP- PP.

### Data collection

Age, clinical manifestations, pathological characteristics, and surgical findings were extracted from the medical charts. The imaging characteristics were obtained after image review.

### Statistical analysis

SPSS 19.0 (IBM, Armonk, NY, USA) was used for statistical analysis. The continuous data were tested for normality test and homogeneity of variances. Normally distributed continuous data are presented as means ± standard deviation, and were analyzed using the Student t test. Skewed continuous data are presented as medians (minimum, maximum), and were analyzed using the Mann-Whitney U test. Categorical data are presented as frequencies and were analyzed using the Fisher’s exact test. Two-sided *P* values < 0.05 were considered statistically significant.

## Results

### Characteristics of the patients

The median age of the 29 patients with SPNP was 30 (range, 12–70) years. The male patients were older than the female patients [median, 56 (28–66) vs. 29 (12–70), *P* = 0.012]. Six of the seven male patients were > 50 years of age. The most common clinical manifestation of the male SPNP patients was abdominal pain or abdominal discomfort (71.4%, 5/7). The clinical presentations and tumor sites were not significantly different between the male and female patients (all *P* > 0.05) (Table 1). The SPNP in all seven male patients were single lesions, with three at the head of pancreas, one at the body of pancreas, and three at the tail of pancreas. The SPNP in the 22 females were also single lesions, with four at the head of pancreas, two at the neck of pancreas, nine at the body of pancreas, and seven at the tail of pancreas. The levels of tumor biomarkers (including CEA and CA19–9), pancreatic enzymes, and blood glucose were all in the normal ranges.

**Table 1:** Comparisons of the clinical manifestations of the male and female patients with SPNP

Clinical manifestation	Males (*n* = 7)	Females (*n* = 22)	*P*
Median age (years)	56.0 (28.0,66.0)	29.0 (12.0,70.0)	0.012
Clinical manifestation			0.872
Abdominal pain or abdominal discomfort	5 (71.4%)	15 (68.2%)	
Physical examinations	2 (28.6%)	7 (31.8%)	
Tumor site			0.904
Head/neck of pancreas	3 (42.9%)	6 (27.3%)	
Body/tail of pancreas	4 (57.1%)	16 (72.7%)	

### MSCT characteristics

**Fig. 1:**
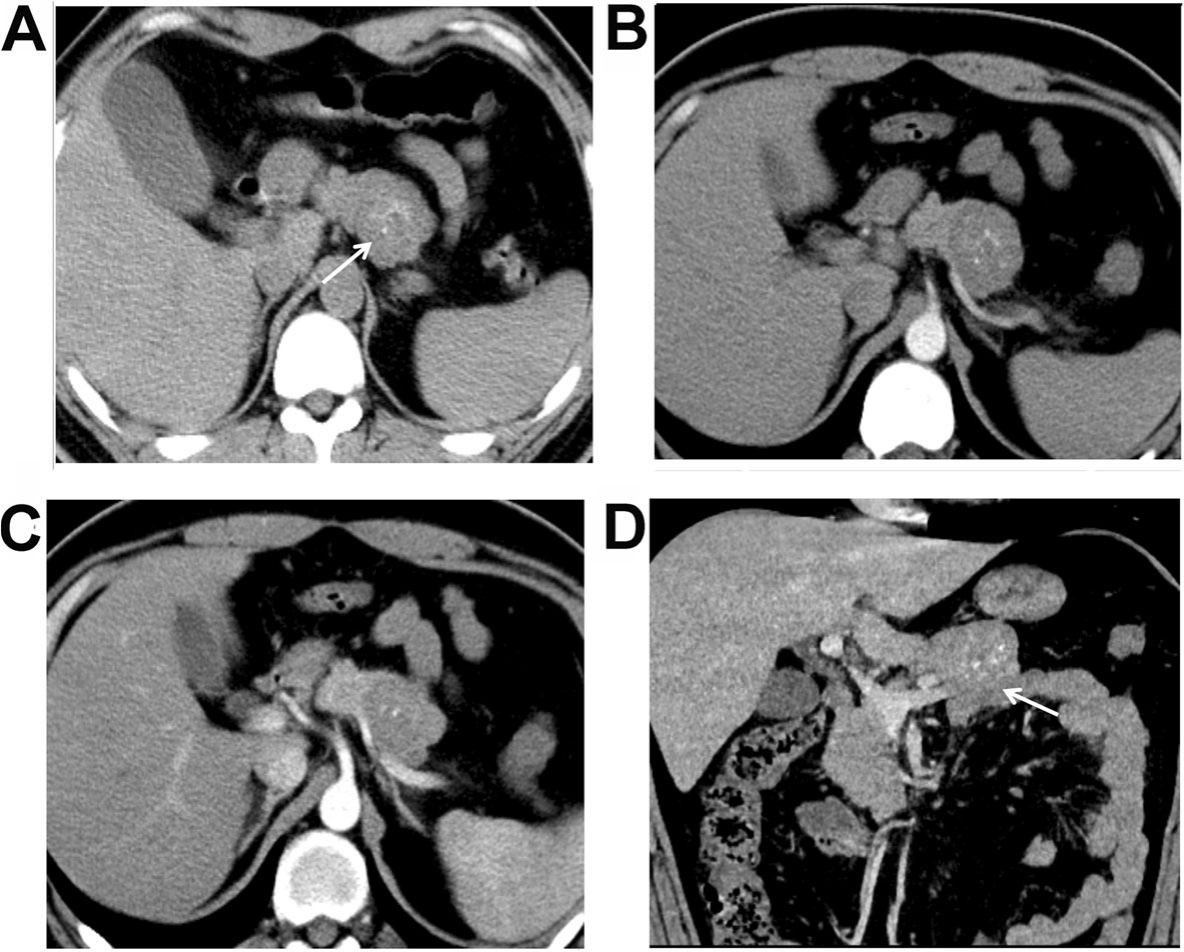
A 28-year-old male patient was found with pseudo-papillary neoplasm of pancreas (SPNP) at physical examinations. **a** An iso-density mass was found at the body of pancreas, and punctate calcifications were found in the mass (white arrow). **b** The enhancement in the arterial phase was not evident. **c** Persistent enhancement was found in the portal venous phase, with the enhancement increased evidently. **d** The boundaries of the tumor were clearly in the reconstructed coronal image (white arrow). No dilation of the pancreatic tube or bile duct was found, while atrophy of the pancreatic tail was shown

**Fig. 2:**
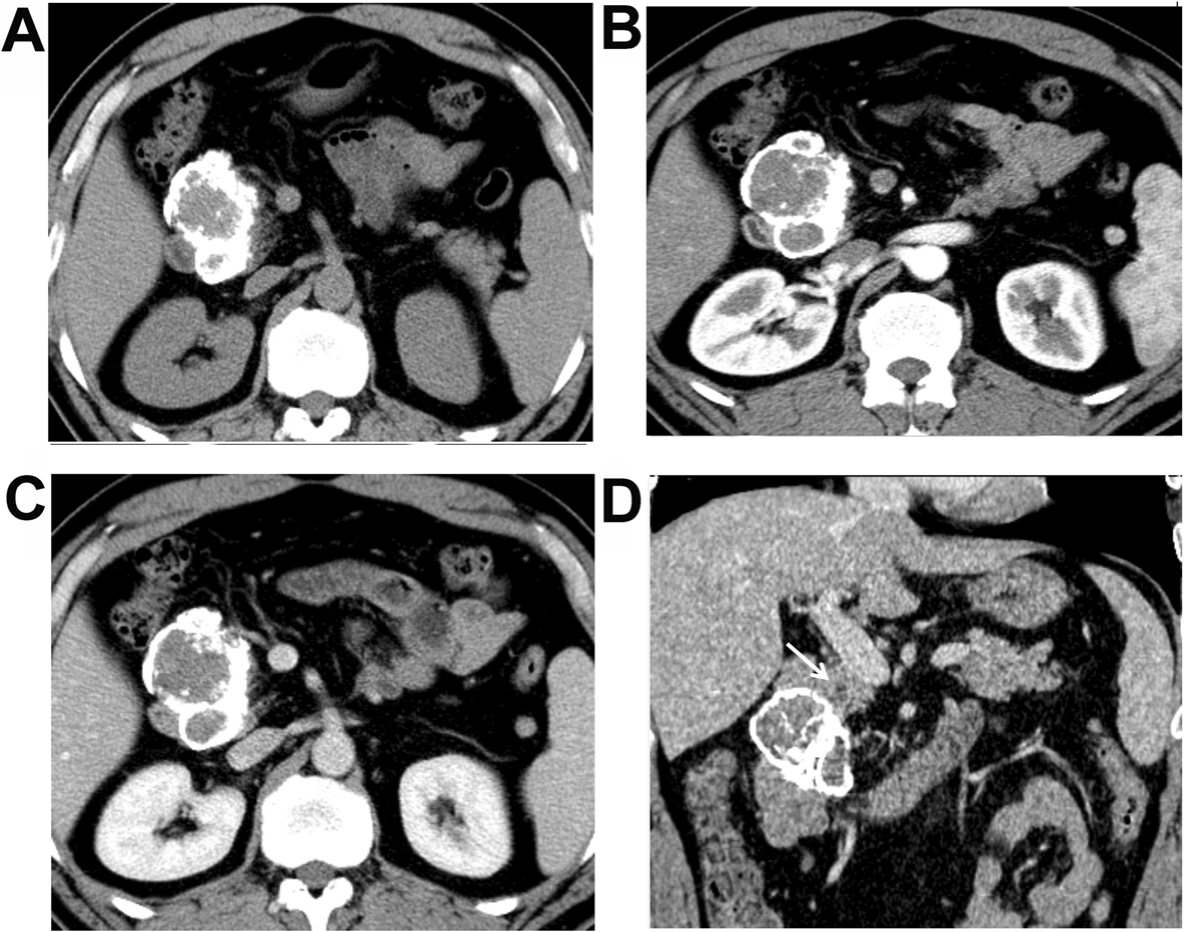
A 49-year-old male patient with discomfort in the upper abdomen. **a** Plain CT scanning + enhanced CT scanning showed pseudo-papillary neoplasm of pancreas (SPNP). **a** Plain scanning showed round solid changes at the pancreatic head, of which the boundaries were clear, and eggshell calcification was shown around the mass. **b** Low enhancement of the lesion in the arterial phase, which was lower than the normal pancreas. **c** Persistent enhancement of the lesion in the portal venous phase was shown. **d** Dilation of the pancreatic tube was shown on the reconstructed coronal image (white arrow)

**Fig. 3:**
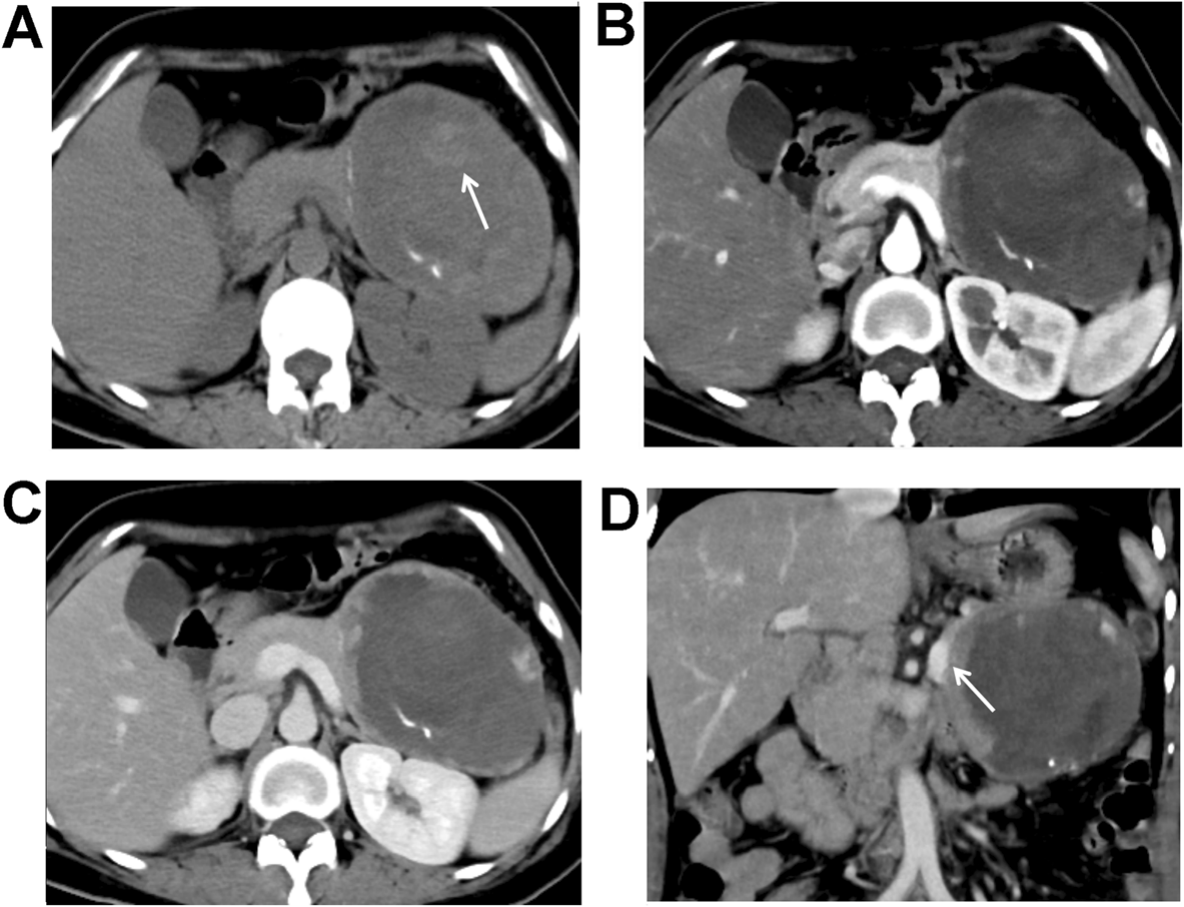
A 50-year-old female patient with pain in the upper abdomen. Plain CT scanning + enhanced scanning showed pseudo-papillary neoplasm of pancreas (SPNP). **a** Plain scanning showed a huge oval cystic and solid mass at the pancreatic tail, of which the boundaries were clear. Punctate calcifications were found around the mass. Small patchy bleeding focuses were found in the mass (white arrow). **b** Low enhancement of the lesion was found in the arterial phase, which was lower than the normal pancreas, and showed “ball-holding” changes with the pancreatic body. **c** Persistent enhancement of the solid components was found in the portal venous phase, which showed “floating cloud” sign. **d** Reconstructed coronal image showed compressing and circuity of the splenic vein (white arrow)

**Fig. 4:**
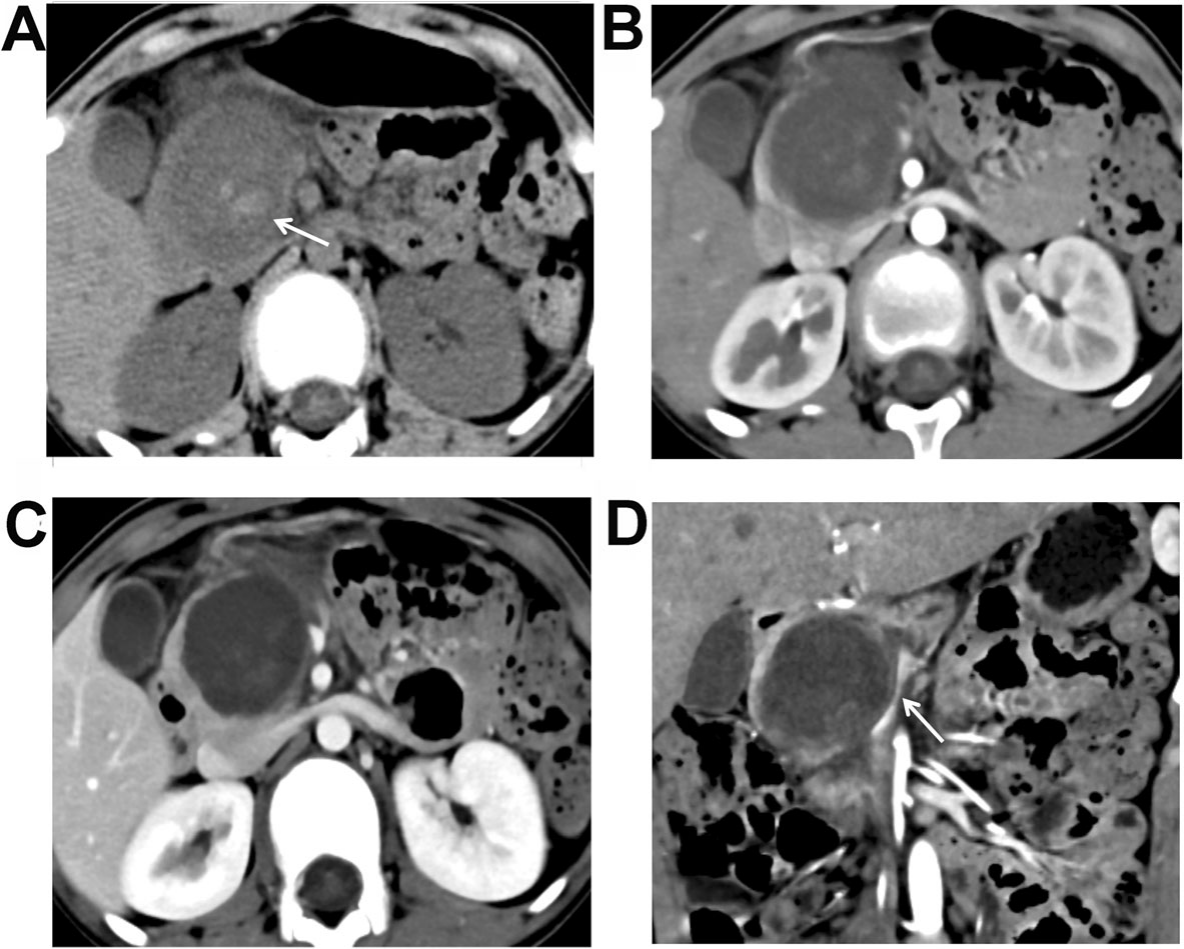
A 12-year-old female patient with vomiting and upper abdominal pain. Plain CT scanning + enhanced scanning showed pseudo-papillary neoplasm of pancreas (SPNP). **a** Plain scanning showed a huge oval cystic mass at the pancreatic head, and the boundaries were clear. Small patchy bleeding focuses were found in the mass (white arrow). **b** Low enhancement of the lesion was found in the arterial phase, which was lower than the normal pancreas. **c** A little enhancement of the cystic components was found in the portal venous phase, which showed “floating cloud” sign. **d** Reconstructed coronal image showed compressing and circuity of the superior mesenteric vein (white arrow)

As shown in Table 2, the median tumor size was 3.9 (range, 2.0–6.4) cm in the male SPNP patients, which was significantly lower than the 7.0 (range, 4.6–14.6) cm in the female patients (*P* < 0.001). The Calcification rate of the SPNP was higher in male than in female patients (*P* = 0.004). In addition, the tumor components were different between the male and female patients of SPNP (*P* = 0.036). Specifically, the percentage of solid tumor was significantly higher in the male patients (85.7%, 6/7), with four patients with completely solid tumors (Fig. 1), and two patients with solid tumors with small cystic changes (cystic components < 10%). The cystic changes in these patients were at the peripheral areas of the tumors. Capsule, bleeding, and enhancement in AP and VP were not significantly different between the male and female patients (all *P* > 0.05). Five of the male SPNP patients were with calcifications in the tumors (71.4%, 5/7), among whom the calcifications were at the center of the lesion in two patients (Fig. 1a-d), at the peripheral of the lesion in two patients, and at the capsule in one patient. One male patient was with eggshell calcification (Fig. 2a-d). For the female patients, seven were found with calcifications, with three at the capsule and four in the tumor (Fig. 3a-d). A capsule was found in 14 female patients, while the boundaries of the capsules were unclear in eight females. Pancreatic duct dilation was found in one female patient and one male patient (Fig. 2d), at the head of the pancreas in both patients. Atrophy of the pancreatic tail was found in one of the 29 patients. The solid components of the tumor in all the 29 SPNP patients showed slight to moderate inflow enhancement. Specifically, slight enhancement in the AP was found, with the enhancement degree lower than the normal pancreatic tissues. Progressive enhancement in the VP and DP was also found, with the area of enhancement increased gradually. The absolute enhancement values in the AP and VP were not significantly different between the male and female groups. The peaked enhancement value appeared in the VP in 15 patients, and in the DP in four patients in the female group. The tumors were chequered with solid components and cystic components in three patients in the female group, which showed the “floating cloud” sign (Fig. 3c).Completely cystic lesions exist only in three female patients (Fig. 4a-d).

**Table 2:** Comparisons of the imaging characteristics of the SPNP

Imaging characteristics	Males (*n* = 7)	Females (*n* = 22)	*P*
Maximum tumor diameter [mean(range)]	3.9 (2.0–6.4)	7.0 (4.6–14.6)	< 0.001
Capsule	5 (71.4%)	14 (63.6%)	0.706
Bleeding	1 (14.3%)	5 (22.7%)	0.484
Calcification	5 (71.4%)	7 (31.8%)	0.013
Tumor component			0.036
Completely solid	4 (57.1%)	2 (9.1%)	
Solid with small cystic changes	2 (28.6%)	6 (27.3%)	
Cystic and solid	1 (14.3%)	11 (50.0%)	
Completely cystic	0	3 (13.6%)	
Mean absolute enhancement value in arterial phase (HU)	23.3 (14.1–50.0)	28.8 (13.2–55.7)	0.413
Mean absolute enhancement value in portal venous phase (HU)	42.8 (23.0–64.3)	45.7 (25.6–70.2)	0.442

### Associations between imaging and pathological findings

Among the 29 patients, the preoperative MSCT suggested SPNP in 21 patients. As shown in Table 3, the diagnostic accuracy of MSCT was higher in the female group than in the male group [77.3% (17/22) vs. 57.1% (4/7)], but the difference was not statistically significant (*P* = 0.360). For the three male patients with unclear preoperative diagnosis, the preoperative MSCT suggested pancreatic cancer or neuroendocrine tumor.

**Table 3:** Diagnostic Accuracy of Preoperative MSCT

	Preoperative Diagnosis	Diagnostic Accuracy
Male(*n* = 7)	4	57.1%
Female(*n* = 22)	17	77.3%

The surgical findings and pathological examinations both showed that the capsule of the SPNP was complete, and the boundaries between tumor and normal pancreatic tissues were clear. The sections of the tumors were solid, chequered with solid and cystic components, or cystic. Microscopy showed that the tumors were with clear margins and covered by a capsule, and the boundaries with the surrounding tissues were clear. The tumor stroma was rich in collagenous fiber and mucinous degeneration. The tumor cells were round or oval, rich of cytoplasm, and were arranged around the blood vessels to show an ependymal-like shape. 

## Discussion

SPNP are rare in men and are often misdiagnosed. Therefore, the aim of this study was to analyze the clinical and MSCT features of patients with SPNP, and examine the differences between males and females. The results suggest that the imaging features of male SPNP are distinct from that of female patients. In males with pancreatic lesions, MSCT generally shows relatively small lesions with higher percentages of solid components and calcification, with typical enhancement suggesting SPNP.

The previous study with the largest sample size [1] reported that SPNP is more common in young females, and that the ratio of males to females was 1:9.78. The mean age of their patients was 22 years. In the present study, median age at presentation was 30 years, and the male to female ratio was 1:3.1. The exact reasons for the age discrepancy could be that the patients seek medical attention later than in the West. Regarding the sex distribution, the bias could be due to the small sample size. In the present study, many male patients were diagnosed by histopathological examination of the surgical specimen. Indeed, as SPNP lacks typical clinical manifestations and as most radiologists are unaware of the imaging presentations in male SPNP patients, the rate of imaging misdiagnosis of SPNP is very high [3].

In this study, the median age of the male SPNP patients was 56 years at, and five of the seven patients were > 50 years of age, which was significantly higher than in the female patients, as supported by Lin et al. [6]. For most of the male SPNP patients (5/7), a capsule was found and the margins were clear, but it was not significantly different from the female SPNP patients. The size of the lesions was smaller in the male SPNP group. These observations indicated that men seem to have a later occurrence of SPNP and suggested that there is a difference in the developmental stage between men and women. In addition, percentage of solid tumors was higher, while percentages of hemorrhagic and cystic changes were lower in the male group than in the female group, which was in agreement with the findings reported by Lam et al. [7]. Zou et al. [14] found that collagen tend to be the main component of tumor stroma in SPNP males,while hyaluronan (HA) composed a considerable proportion in females, which was consistent with the conventional characteristics of SPNP [16]. McCarthy et al. [17] reported that HA in stroma could promote tumor cell proliferation,which may explain the growth pattern and the degenetive changes in females in our study. MSCT scanning showed punctate calcifications or striped calcifications in the SPNP lesions. The calcifications in the tumor could be caused by the degeneration of tumor and deposition of calcium. Calcification could appear in the tumor or at the capsule. Previous studies reported that about 30% of the SPNPs are found with calcification [1, 18]. In this study, we found that the percentage of calcification was as high as 71.4% in the male SPNP patients, which was significantly higher than the 31.8% in the female patients. The difference could be associated with female hormones, as estrogens have been shown to play a role in the development of SPNP [13]. Researchers has reported that a much stronger expression of androgen receptor (AR) was found in males [14], and he also found mutations of CTNNB1 exon 3 was observed in all 30 cases, which distributed at codon 32,33 and 37 in both genders and 34,31 and 62 in females, and this might be a clue to the underlying mechanism of the gender difference. In addition, the relatively sample size of this study could also play a role. Anil et al. [5] and Choi et al. [19] suggested that the rich blood vessels and small blood sinuses in the solid components of the tumor could contribute to the high propensity of hemorrhage. In this study, hemorrhage was found in the SPNPs in both males and females, but the difference between the two groups was not significant. In addition, the capacity of CT of displaying hemorrhage is lower than that of magnetic resonance imaging [20, 21], and thus further examinations were needed for the final diagnosis.

The interior components of SPNP could be solid dominant, cystic and solid, and cystic dominant. For the cases with solid dominant SPNP, the cystic components could be covered by the capsule. For the cases with cystic and solid SPNP, the tumor could show a “floating cloud” sign that the solid components seemed to float in the cystic components, or the tumor was chequered with solid components and cystic components [5]. While for the cystic dominant SPNP, the solid components were mainly shown as the mural nodules. The enhancement pattern of typical SPNP is low enhancement in the AP, with the enhancement degree lower than the normal pancreatic tissues; while the VP and DP show progressive enhancement, with the area of enhancement increase gradually [5, 21, 22]. In this study, the pattern and degree of enhancement in the male patients were similar to the typical SPNP. The interior components of SPNP, as well as the enhancement pattern, were not significantly different between the male and female patients. Nevertheless, no “floating cloud” sign was found in the male SPNP patients, which could be associated with the lesser extent of cystic changes in the male patients. A previous study has reported that relatively large tumor at the pancreatic head could compress the pancreatic and bile ducts, and induce the dilation of such ducts, but would not induce the atrophy of the distal end of pancreas [12]. In this study, nine patients were found with the SPNP at the pancreatic head, among which two were found with dilation of the pancreatic and bile ducts, and one was with atrophy of the pancreatic tail, which was in agreement with the findings reported by Hu et al. [3]. We speculated that such changes could be associated with the relatively slow growth of the tumor, or the neuroendocrine origin tumors which were mainly in the pancreatic parenchyma and tended to grow outward [23]. Therefore, the pancreatic ducts and the surrounding structures were generally compressed.

The present study has limitations. First, it was a retrospective study of a rare condition and conducted in a single center. Hence, the sample size was small. A porspective study is needed to validate the results in multiple centers. Second, there is no genetic and molecular level of auxiliary experiments, and so it is not possible to clarify the causal relationship between genetic mutations and MSCT characteristics. Therefore, it is necessary to expand the statistical samples, and improve the study on gene and molecular level.

## Conclusions

In summary, SPNP is rare in male patients. The clinical and imaging features of SPNP are different between male and female patients. Specifically, the male SPNP patients are older and the tumors are smaller. In addition, the tumors contain higher percentage of solid components and calcifications. SPNP should be considered for male patients with pancreatic mass.

## Data Availability

The datasets used and/or analyzed during the current study are available from the corresponding author on reasonable request.

## Abbreviations

AP: Arterial phase
CT: Computed tomography
DP: Delayed phase
MSCT: Multi-slice computed tomography
PP: Plain phase
ROI: Region of interest
SPNP: Solid-pseudo papillary tumor of pancreas
VP: Venous phase
